# Role of Various Commercial *Saccharomyces cerevisiae* in Secondary Fermentation of Pineapple Glutinous Rice Wine: Insights into Volatile Profiling by GC-IMS and Sensory Attributes

**DOI:** 10.3390/foods15122238

**Published:** 2026-06-21

**Authors:** Derong Ji, Zhiyue Ge, Shuheng Zou, Lili Duan, Dujie Liu, Pei Xu, Mingfeng Qiao

**Affiliations:** 1 Cuisine Science Key Laboratory of Sichuan Province, Sichuan Tourism University, Chengdu 610100, China; jdr1001@163.com (D.J.); liliduan2026@163.com (L.D.); xupei386@gmail.com (P.X.); 2 College of Culinary and Food Science Engineering, Sichuan Tourism University, Chengdu 610100, China; zyge1120@163.com (Z.G.); u2974500075@163.com (S.Z.); ldj29011401@163.com (D.L.)

**Keywords:** pineapple glutinous rice wine, *Saccharomyces cerevisiae*, fermentation characteristics, quality influence

## Abstract

Pineapple glutinous rice wine is a popular fermented beverage, yet its flavor and quality remain highly affected by yeast strains. This study aimed to explore the effects of four commercial yeasts, namely La Raffinée (Ra), La Bayanus (Ba), La Délicieuse (De), and Angel wine yeast (AWY), on the quality of pineapple glutinous rice wine. Fermentation indices including Brix and alcohol content were dynamically monitored, while final wine samples were analyzed for color difference, sensory quality and aroma components by gas chromatography–ion mobility spectrometry (GC-IMS). Results showed that all yeasts had optimal fermentation efficiency, sugar-to-alcohol conversion, physicochemical properties and sensory quality at an addition level of 0.2 g/L. The 0.2 g/L De group achieved the optimal overall quality with an alcohol content of 10.01% vol, the highest ester content (22.55%) and the lowest acid content (12.18%), presenting a balanced flavor, prominent pineapple aroma and refreshing taste. In contrast, Ba led to higher acidity and Angel wine yeast contributed to greater sweetness. Overall, 0.2 g/L De yeast effectively coordinated pineapple aroma and glutinous rice wine characteristics, providing a practical reference for optimizing the production and quality improvement of pineapple glutinous rice wine.

## 1. Introduction

*Saccharomyces cerevisiae* is the predominant microorganism in the winemaking fermentation process and serves as the primary factor influencing the aroma components of wine. It has a decisive impact on the quality and flavor of wine samples [1]. Commercial yeasts are characterized by high stability and specific fermentation attributes; they effectively enhance fermentation efficiency, improve product quality, and reduce fermentation costs. Common commercial yeast brands include Angel, Diboshi, Holy Family, and Schuckleman [2]. The utilization of commercial yeasts helps ensure product consistency and repeatability. As a typical low-alcohol fermented beverage, glutinous rice wine is a low-alcohol fermented liquor produced through saccharification and fermentation using glutinous rice as the main raw material and koji as the saccharifying agent. Compound fruit wine combines the effects of two or more fruits, resulting in a product with good color, mellow taste, typical flavor, and full-bodied profile [3]. Pineapple (Ananas comosus) is the third-largest tropical fruit, following bananas and citrus fruits. It is native to the tropical and subtropical regions of South America and is widely cultivated in Guangdong, Guangxi, and Fujian provinces of China [4,5]. Pineapple juice is rich in nutrients, containing abundant sugars, proteins, vitamins, organic acids, and flavonoids. It offers various health benefits, such as strengthening the stomach and stimulating appetite, tonifying the spleen and alleviating diarrhea, as well as clearing the stomach and quenching thirst. Furthermore, pineapple flavor is one of the common flavors found in beverages [6].

Aroma composition is the core index to evaluate the sensory quality and market acceptance of pineapple glutinous rice wine. In this study, Gas Chromatography–Ion Mobility Spectrometry (GC-IMS) was used for volatile compound analysis, rather than conventional techniques. IMS shows higher sensitivity for trace volatile flavor substances, enables rapid and real-time detection with simple sample preparation, and is more suitable for volatile profiling in complex matrices like pineapple glutinous rice wine with diverse components and matrix interference. To investigate the effects of yeast strains on the quality of pineapple glutinous rice wine, we optimized the traditional fermentation process to reduce the interference of sweet wine koji on the fermentation processes of different yeasts. Four representative commercial yeasts were chosen based on their wide application in fruit wine and glutinous rice wine fermentation. Ra, Ba, and De are imported specialty yeasts with stable fermentation performance and excellent flavor-producing capacity; AWY is a domestic high-activity dry yeast with strong adaptability in low-alcohol rice wine systems. Together they enable a comprehensive comparison of yeast effects on wine quality. Physicochemical indicators of the wine samples were determined and monitored dynamically during and after fermentation. Subsequently, we compared the effects of these yeast strains on wine quality. This study provides practical references for the process optimization and quality improvement of pineapple glutinous rice wine.

## 2. Materials and Methods

### 2.1. Materials and Instruments

Perfume pineapples were sourced from Xuwen, Zhanjiang City, Guangdong Province. Glutinous rice, characterized by its distinct grain flavor, originated from Wuchang, Heilongjiang Province. Four types of active dry yeasts for fruit wine were utilized: La Raffinée (Ra), La Bayanus (Ba), La Délicieuse (De) and Angel yeast (Angel wine yeast; AWY). These yeasts were provided by Yantai Diboshi Brewing Co., Ltd. (Yantai, China) and Angel Yeast Co., Ltd. (Yichang, China). Food-grade white sugar was supplied by Yunnan Wangyi Agricultural Science and Technology Development Co., Ltd. (Kunming, China), while pectinase (30,000 U/g) was obtained from Shandong Longkete Enzyme Preparation Co., Ltd. (Linyi, China).

Instruments utilized in this study included the GL2241-1SCN-type electronic balance (Shenzhen Lintao Instrument Co., Ltd., Shenzhen, China), the CJM-2474 alcohol meter (Hebei Hengshui Micromei Co., Ltd., Hengshui, China), the UV-7504 UV-visible spectrophotometer (Anhui Ninghuai Instrument Co., Ltd., Tianchang, China), the BM-03 sugar meter (Tianjin Outlook Photoelectric Technology Co., Ltd., Tianjin, China), the SC-80C color difference meter (Hangzhou Color Spectrum Technology Co., Ltd., Hangzhou, China), the FlavourSpec^®^ Gas Chromatography–Ion Mobility Spectrometry (GC-IMS) combined instrument (G.A.S. GmbH, Dusseldorf, Germany), and the DHP-9052 constant temperature incubator (Shanghai Yiheng Scientific Instrument Co., Ltd., Shanghai, China).

### 2.2. Experimental Methods

#### 2.2.1. Production Process and Key Points of Pineapple Glutinous Rice Wine

Rice wine preparation: Rinse glutinous rice → Soak it → Steam it → Allow it to cool → Begin Fermentation I (incorporate sweet rice koji) → Sterilize (once Fermentation I is finished).

Pineapple rice wine preparation: Treat pineapple juice with pectinase → Mix (mix rice wine and pineapple juice in a 1:1 ratio) → Adjust sugar content (add white sugar) → Fermentation II (add commercial yeast) → Filter → Bottle → Sterilize → Obtain the finished product.

Operation details: Wash the glutinous rice and soak it for 6 h. Cook the glutinous rice for 40 min until the white grains in the rice disappear. Allow the cooked glutinous rice to cool to 30 °C, then add 10 g/kg of sweet wine koji and stir evenly. Add an appropriate amount of distilled water, seal the fermenter, and ferment the rice grains at 30 °C for 2 days. After fermentation, filter the resulting rice wine to remove the rice grains, and set the filtrate aside for later use. Peel the pineapple, extract the juice, and add 0.1 g/L of pectinase; then hydrolyze at 30 °C for 120 min before use. This condition was optimized by preliminary experiments to achieve higher juice yield and better clarification. Mix the rice wine and pineapple juice in a 1:1 ratio to achieve a total sugar content (measured as glucose) of 221 g/L. Prepare 12 groups of experimental samples for Ra, Ba, De, and AWY with concentration gradients of 0.1 g/L, 0.2 g/L, and 0.3 g/L. Place these samples in a 36 °C water bath for 30 min for heat preservation and activation, stirring constantly [7]. Finally, ferment the samples at 25 °C, then filter and sterilize the fermented products to obtain the final samples. The control group (M) was pure glutinous rice wine without pineapple juice and secondary yeast fermentation.

#### 2.2.2. Determination of Basic Physical and Chemical Indices

The alcohol content was determined according to the national standard GB/T 13662-2008 “Chinese Rice Wine” [8]. The content of soluble solids (sugar degree) was measured using a sugar meter. The total sugar content of pineapple glutinous rice wine was determined with reference to Liu Zhongyi’s 3,5-dinitrosalicylic acid method [9]. According to the national standard GB/T 12456-2021 “Determination of total acid in food” [10], the acid-base titration method was used to determine the total acid content of the sample. The L*, a*, b* values and ΔE* values of each sample were measured by a color difference meter to determine the total color difference.

#### 2.2.3. Determination of Total Flavonoids

The Al(NO_3_)_3_-NaNO_2_-NaOH color-development method [11] was adopted. Referring to Ji Junchen [12], a rutin standard curve was prepared. The standard curve was plotted with the concentration of the rutin standard solution as the abscissa and the light absorption value as the ordinate, and the regression equation obtained was y = 0.0518 x + 0.0144 (R^2^ = 0.9914).

#### 2.2.4. Determination of VC Content

The method of VC determination by Wang Chuanfen [13] was referred to.

#### 2.2.5. Sensory Assessment

In accordance with the ISO 8586:2012 [14] standard, the final sensory evaluation panel consisted of 6 male and 6 female panelists majoring in winemaking. This panel was expanded from the 10 assessors initially adopted; two additional qualified evaluators were newly recruited after standardized screening and training. The original sensory data of the initial 10 panelists were completely retained and integrated with the datasets of the two newly supplemented members for subsequent statistical analysis, instead of repeating the entire sensory experiment. All panelists were strictly screened before the test: they had normal taste and olfactory functions, no smoking habits, no nasal or oral diseases, and had basic experience in fruit wine sensory evaluation. Before formal scoring, all panelists received systematic calibration training using standard gradient reference solutions corresponding to pineapple aroma, rice wine aroma, floral aroma, sweetness, and acidity. Meanwhile, special repeated training for the 10-point intensity scale was conducted to unify the scoring criteria, ensuring that all panelists could stably and reproducibly judge sensory intensity. All panelists were capable of accurately describing the aroma properties of the samples corresponding to the aroma intensity levels of fruit wines. The team members quantified the pineapple aroma, rice wine aroma, floral aroma, sweetness, acidity, taste balance, and aroma richness of different yeast-added and different-type pineapple glutinous rice wines on a scale from 1 (low intensity) to 10 (high intensity). For each sample group, the mean score of each sensory attribute was calculated from all panelists’ ratings. One-way ANOVA was performed to evaluate panel performance, and the results showed no significant differences in scores among different panelists (*p* > 0.05), indicating excellent inter-assessor reliability and stable sensory evaluation performance [15].

#### 2.2.6. Volatile Substances Analysis (GC-IMS)

The volatile compounds in pineapple glutinous rice wine were analyzed using a FlavourSpec^®^ GC-IMS instrument (G.A.S. GmbH, Germany), following the method of reference [16] with minor modifications. Briefly, 5 mL of sample was transferred into a 20 mL headspace vial. The automatic headspace injection parameters were set as follows: incubation temperature of 60 °C, incubation time of 10 min, incubation speed of 500 rpm, and injection volume of 200 μL. The separation of volatile compounds was performed on a MXT-WAX (30 m × 0.53 mm × 1 μm, RESTEK, Mount Ayr, IA, USA), with the column oven temperature maintained at 60 °C throughout the 30-min analysis. High-purity nitrogen (99.99%) was used as the carrier gas, with a programmed flow rate gradient: 2 mL/min for initial 5 min, linearly increased to 10 mL/min for 10 min, 15 mL/min for 5 min, 50 mL/min for 10 min, and 100 mL/min for 10 min. For ion mobility spectrometry, high-purity nitrogen (99.99%) served as the drift gas at a constant flow rate of 150 mL/min, with the drift tube temperature maintained at 45 °C. The retention indices (RIs) of volatile compounds were calculated using n-ketones (C4–C9) as external references. Compounds were tentatively identified by comparing their RIs and ion drift times with those of standards in the GC-IMS library.

### 2.3. Data Processing and Analysis

All experiments were performed in triplicate. Data were processed using Microsoft Excel 2016. Correlation analysis and significance difference analysis were evaluated and processed with SPSS 26.0 statistical software. One-way ANOVA followed by Duncan’s multiple range test was used for multiple comparisons, and significance was defined at *p* < 0.05. Flavor substance heat map analysis was conducted using Origin 2021.

## 3. Results

### 3.1. Quality Changes in Wine Samples During Fermentation

**Figure 1:**
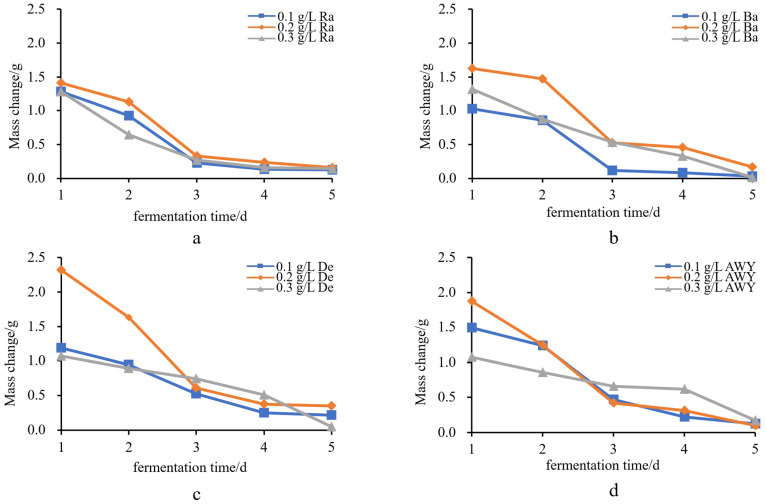
Changes in the quality of wine samples during the fermentation process of different varieties of commercial yeast. (**a**): Wine fermented with De (0.1, 0.2, 0.3 g/L); (**b**): Wine fermented with Ra (0.1, 0.2, 0.3 g/L); (**c**): Wine fermented with Ba (0.1, 0.2, 0.3 g/L); (**d**): Wine fermented with AWY (0.1, 0.2, 0.3 g/L).

Carbon dioxide (CO_2_) is one of the main products of yeast fermentation. Thus, the mass loss during the fermentation process can effectively reflect the fermentation efficiency [17]. The CO_2_ weight loss method is commonly used to represent the fermentation capacity of yeast, that is, the alcohol-producing capacity of yeast, which is an important indicator for selecting high-quality yeast [18]. Figure 1a–d illustrates the quality changes in wine samples under different concentrations of Ra, Ba, De, and AWY, respectively. The quality of the wine samples gradually showed a downward trend as the fermentation time increased. On the first and second days of fermentation, the quality of the wine samples fermented by the four types of yeasts changed relatively rapidly. It can be inferred that the intensity of yeast respiration and fermentation efficiency were significantly enhanced on the first and second days of fermentation. On the 4th and 5th days of fermentation, the quality of the wine samples did not change significantly, indicating that the fermentation of the wine samples was basically completed.

When the yeast addition level was 0.2 g/L, the total quality change in the four yeast samples during fermentation was higher than that of the samples with 0.1 g/L and 0.3 g/L yeast addition levels. The quality change values of the 1-day and 2-day samples were significantly higher than those of the samples with 0.1 g/L and 0.3 g/L yeast addition levels. Among the four types of yeasts, the sum of the quality change values of the wine samples fermented by 0.2 g/L De yeast was the largest, reaching 5.29 g, which was 61% larger than the sum of the quality change values of the 0.2 g/L Ra samples, the smallest among them. It can be preliminarily concluded that the fermentation effect is better when the yeast addition amount is 0.2 g/L. The fermentation effects of 0.2 g/L Ba and AWY wine samples are similar, while the fermentation effect of 0.2 g/L Ra yeast wine samples is relatively poor.

### 3.2. Changes in Alcohol and Sugar Content During Fermentation

**Figure 2:**
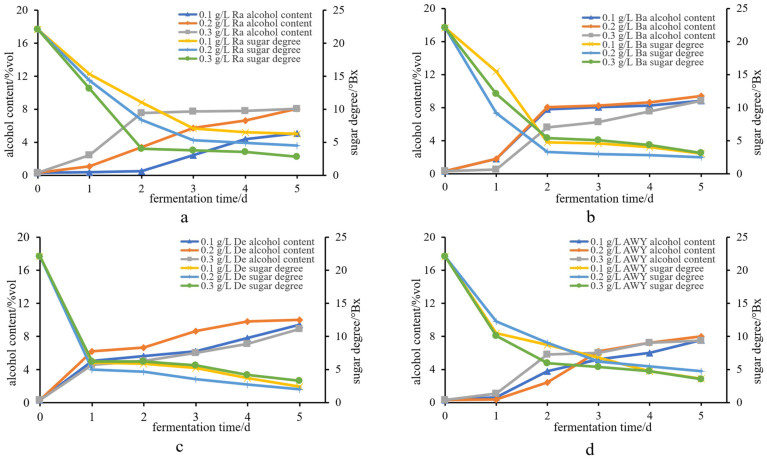
Results of alcohol content and sugar content after fermentation with different varieties of commercial yeast. (**a**): Wine fermented with De (0.1, 0.2, 0.3 g/L); (**b**): Wine fermented with Ra (0.1, 0.2, 0.3 g/L); (**c**): Wine fermented with Ba (0.1, 0.2, 0.3 g/L); (**d**): Wine fermented with AWY (0.1, 0.2, 0.3 g/L).

The changes in alcohol content and sugar content are directly influenced by the type and amount of yeast added. Excessive yeast addition may cause the fruit wine to become cloudy and have a strong yeast taste [19], while insufficient addition may affect the fermentation efficiency and lead to an unstable balance in the wine body and taste. The changes in alcohol content and sugar content of Ra, Ba, De, and AWY at different concentrations were analyzed to further evaluate the fermentation effect of the yeasts. The specific results are presented in Figure 2a–d. The alcohol content showed an upward trend on the first and second days of fermentation, while the sugar content showed a downward trend. The change slope of the alcohol content was significantly smaller than that of the sugar content, and the change amplitudes of the two were inconsistent, which might be due to the consumption of part of the fermented sugar required for yeast growth [20].

The alcohol content of the four yeast-fermented samples was higher and the sugar content was similar when the yeast addition was 0.2 g/L. Under this dosage, the sugar-to-alcohol conversion rate of Ra was 56.50%, that of Ba was 65.80%, that of De was 70.62%, and that of AWY was 56.01%. The wine sample fermented by De had the highest alcohol content (10.01% vol), the lowest sugar content (2.03 °Bx), and the highest sugar-to-alcohol conversion rate (70.62%). It can be concluded that, at the same yeast addition level, De utilizes sugar more fully than Ra, Ba, and AWY. The alcohol content and taste balance of the wine are directly determined by the sugar-acid ratio of the brewing raw materials [5]. Therefore, based on the changes in the alcohol content and sugar content of the wine samples, it can be judged that the 0.2 g/L De yeast has the best fermentation effect, followed by the Ba yeast, which is inferior to De but superior to Ra and AWY. The conversion rates of Ra and AWY are close, and their fermentation effects are relatively poor.

### 3.3. Total Sugar Content

**Figure 3:**
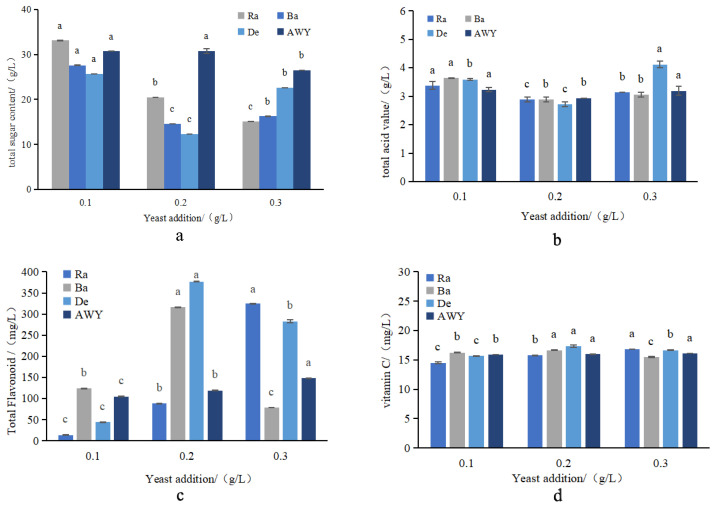
Determination Results of Key Physicochemical Indicators in Pineapple Glutinous Rice Wine fermented by different commercial yeasts with addition levels of 0.1 g/L, 0.2 g/L and 0.3 g/L. Note: a, b and c represent the significance comparison results of samples prepared with different dosages of the same yeast strain. (**a**) refers to the total sugar content, (**b**) refers to the total acid content, (**c**) refers to the total flavonoid content, (**d**) refers to the vitamin C content.

The total sugar content is a crucial indicator for evaluating the fermentation effect of wine samples [21]. As depicted in Figure 3a, both the yeast type and the yeast addition amount had an impact on the total sugar content of the wine samples. Generally, the total sugar content of the wine samples was higher at 0.1 g/L than at 0.2 g/L and 0.3 g/L. The total sugar content of Ba and De wine samples first decreased and then increased with the increase in yeast addition. For Ra samples, the total sugar content showed a decreasing trend, with the highest value of 33.12 ± 0.03 g/L (*p* < 0.05) in 0.1 g/L Ra samples and the lowest value of 12.29 ± 0.03 g/L (*p* < 0.05) in 0.2 g/L De samples, which was 2.7-fold lower than that of 0.1 g/L Ra samples. The total sugar content of 0.1 g/L Ra samples was twice as high as that of 0.3 g/L yeast-added samples. The total sugar content in the finished wine samples of AWY decreased significantly at 0.3 g/L, indicating that the yeast addition amount had a significant effect on the total sugar content of the wine samples. In summary, different yeast types and addition amounts affected the fermentation effect of pineapple glutinous rice wine.

### 3.4. Total Acid Content

Total acid refers to the sum of the concentrations of both ionized and un-ionized hydrogen ions in fruit wines. It serves as an important index for assessing the normality of the fermentation process [22,23]. Figure 3b illustrates the total acid content of wine samples fermented with different commercial yeasts. The data exhibited a decreasing trend followed by an increasing trend with the addition of yeast. Specifically, the total acid content of wine samples with 0.2 g/L yeast addition was lower than that of 0.1 g/L and 0.3 g/L. The wine sample fermented with 0.2 g/L De had the lowest total acid content at 2.71 ± 0.08 g/L (*p* < 0.05), whereas the sample fermented with 0.3 g/L De had the highest total acid content, reaching 4.11 ± 0.12 g/L (*p* < 0.05). During the fermentation of fruit wines, yeast participates in the metabolism of various organic acids, such as malic and citric acid, which reduces the acidity of the wine. However, an excessive amount of yeast may lead to increased acid production [24]. Additionally, the amount and type of yeast added can influence the metabolism and synthesis of acids, resulting in variations in the organic acid content of the finished products. In conclusion, both the concentration and type of yeast significantly impacted the total acid content in the final products, with a 1.52-fold difference observed between the total acid content of 0.2 g/L De and that of 0.3 g/L De. The acid contents of the wine samples fermented by Ra, Ba, and AWY were relatively similar, and the fermentation effects of the four types of yeasts were all moderately effective.

### 3.5. Color Difference

According to the CIE Lab color system, L* represents the lightness of color, a* is related to the red component, and b* is related to the yellow component. A higher L* value indicates a lighter-colored sample, a lower a* value means a smaller red component, and a lower b* value implies a smaller yellow component in the wine color [25]. When comparing the L* values of the same yeast with different additions, significant differences were observed overall. According to Table 1, the L* value of 0.2 g/L De samples (13.41 ± 0.06, *p* < 0.05) was significantly higher than that of 0.1 g/L, 0.3 g/L De samples, and samples of other different yeast types and additions (*p* < 0.05), suggesting that the 0.2 g/L De samples had a lighter and clearer color. Additionally, the a* value of 0.2 g/L De was significantly lower than that of 0.1 g/L, 0.3 g/L De and other different yeast species and additions, with a value of 4.14 ± 0.04 (*p* < 0.05), resulting in a less obvious red color. The b* value of 0.2 g/L De was higher than that of 0.1 g/L, 0.3 g/L De and other different yeast species and additions, at 14.30 ± 0.18 (*p* < 0.05), indicating a higher yellow fraction and a more distinct color. The color difference (ΔE*) is related to the contribution degrees of L*, a*, and b* values and characterizes the overall degree of variation among wine samples [25]. A comparison of the difference analysis of the four yeasts with different additions of the same yeast showed that the yeast additions had a significant effect on the color difference. The ΔE* value of 0.2 g/L De was the smallest compared with that of 0.1 g/L, 0.3 g/L De, and other different types of yeasts and additions, which was 20.04 ± 0.12 (*p* < 0.05), indicating that the overall color was balanced, and the degree of color difference was relatively small.

### 3.6. Flavonoid Content and VC Content

Pineapple is rich in flavonoids and vitamin C. Flavonoids, as polyphenols, are physiologically active substances with antiradical, antioxidant, and bacteriostatic effects [26], while vitamin C serves as both an antioxidant and a coenzyme, which can prevent the body from being oxidized by free radicals [27]. The total flavonoid content of pineapple glutinous rice wine measured at the beginning of fermentation was 692.73 ± 0.46 mg/L (*p* < 0.05), and the VC content was 18.99 ± 0.02 mg/L (*p* < 0.05). The results of the total flavonoid and vitamin C content of the fermented products are presented in Figure 3c and Figure 3d, respectively. A comparison of the total flavonoid content of wine samples obtained from the same yeast with different additives revealed significant differences in the flavonoid content of the finished products (*p* < 0.05), indicating that the yeast additions had a significant effect on the total flavonoid content of wine samples. The 0.2 g/L De sample had the highest total flavonoid content of 377 ± 0.46 mg/L (*p* < 0.05), while the total flavonoid contents of 0.2 g/L Ba and 0.3 g/L Ra were similar. The three AWY-supplemented groups exhibited relatively low total flavonoid levels. The total flavonoid content of 0.3 g/L Ba samples was significantly lower than that of 0.1 g/L and 0.2 g/L Ba, with a value of 78.83 ± 0.46 mg/L (*p* < 0.05). The total flavonoid content of all samples was lower than that before fermentation, indicating that the type and concentration of the yeast affected the retention degree of antioxidants in the samples.

From Figure 3d, it can be seen that there was a significant difference (*p* < 0.05) in the vitamin C content of the finished wine samples fermented with different commercial yeasts, indicating that the amount of yeast added had a significant effect on the vitamin C content. Among the four yeasts, there were differences in vitamin C content. The 0.2 g/L De-fermented wine sample had the highest vitamin C content of 17.35 ± 0.21 mg/L (*p* < 0.05), while the 0.1 g/L Ra-fermented wine sample had the lowest vitamin C content of 14.49 ± 0.14 mg/L (*p* < 0.05). The vitamin C contents of all wine samples were lower than that in the pre-fermentation samples, indicating that different commercial yeasts had a certain impact on the retention of vitamin C in the fermented wine samples.

The best retention of flavonoids and vitamin C was observed in the 0.2 g/L De yeast, followed by the Ba and AWYs, and the worst retention of vitamin C and flavonoids was observed in the 0.1 g/L Ra yeast. This indicates that 0.2 g/L De had the best retention of antioxidants among the four yeasts. In conclusion, the type and amount of yeast added significantly affected the retention of antioxidants in wine samples. Combined with the results of the correlation analysis in Table 2, the amount of yeast added had a significant effect on the three factors of total sugar, vitamin C, and flavonoids in the wine samples, with highly significant negative correlations with total sugar (*p* < 0.01), significant positive correlations with vitamin C (*p* < 0.05), and highly significant positive correlations with flavonoids (*p* < 0.01).

### 3.7. Sensory Analysis

**Figure 4:**
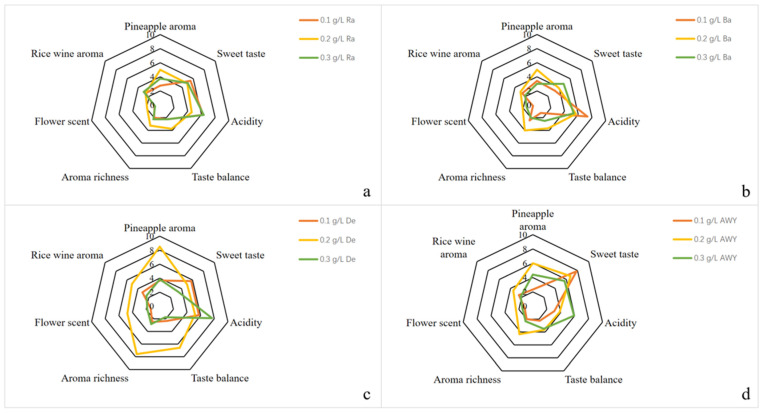
Sensory evaluation of wine samples fermented by different commercial yeasts. (**a**): Wine fermented with De (0.1, 0.2, 0.3 g/L); (**b**): Wine fermented with Ra (0.1, 0.2, 0.3 g/L); (**c**): Wine fermented with Ba (0.1, 0.2, 0.3 g/L); (**d**): Wine fermented with AWY (0.1, 0.2, 0.3 g/L).

The sensory analysis results of wine samples after fermentation with different commercial yeasts are presented in Figure 4. The acidity of the wine samples fermented by Ra and Ba yeasts was more prominent, resulting in a lower taste balance. It may be necessary to reduce the acidity to improve the taste balance. At the same time, the characteristic aromas of pineapple, rice wine, and floral aroma were less obvious, leading to a low degree of aroma richness. Among the three concentrations of De yeast, the 0.2 g/L addition had the most distinct characteristic aroma, the highest aromatic richness, moderate sweetness and sourness, and a better taste balance. In contrast, the 0.1 g/L and 0.3 g/L additions had lower aromatic richness compared with 0.2 g/L, and the 0.3 g/L addition had a more obvious sourness, which affected the taste balance. The samples fermented by the AWY had a more obvious sweetness, which required acid treatment to improve the taste balance. At the same time, the characteristic aroma was not obvious, and the overall aromatic richness was lower. Overall analysis showed that the fermentation effect of 0.2 g/L De was optimal, and the quality of the wine samples was relatively the best.

### 3.8. GC-IMS Determination

#### 3.8.1. Analysis of Volatile Matter Composition

**Figure 5:**
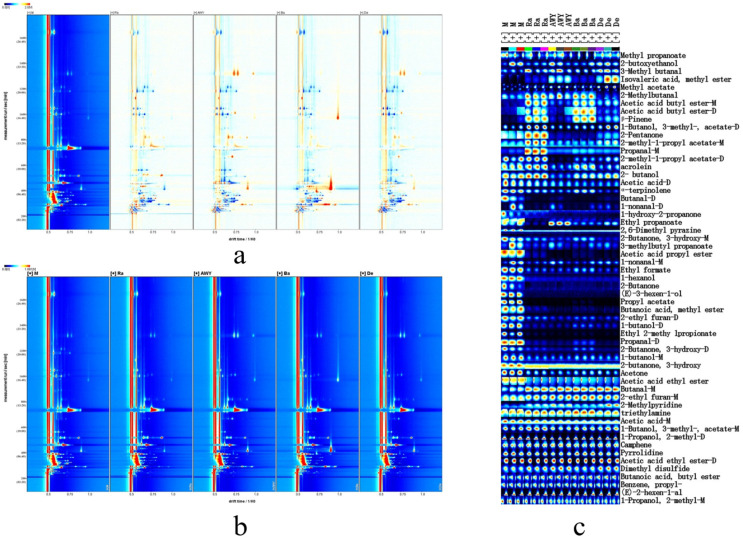
Volatile Substance Analysis of Pineapple Glutinous Rice Wine Fermented by Different Commercial Yeasts: (**a**) refers to the volatile compounds in wine samples; (**b**) refers to the differences in volatile compounds in wine samples; (**c**) refers to the Gallery Plot fingerprint spectra of wine samples.

The volatile substance compositions in different wine samples are shown in Figure 5a. The drift time of 0.5 ms represents the reactive ion peak, and each point on both sides of the peak indicates a substance. The shade of red color reflects the content of the substance [28]. The GC-IMS analysis revealed significant differences in the types and contents of substances in the five samples. The differences in volatile substance components in different wine samples are presented in Figure 5b. To compare the differences in substance types and contents in different yeast-fermented samples, pure glutinous rice wine (marked as M, control group) was used as a reference. The remaining four samples were plotted after deducting the reference, and the background of the four commercial yeast samples after background deduction was presented in white. In the graph, a red color indicates that the substance concentration is higher than that of pure rice wine, and a darker blue color indicates that the concentration of the substance is lower than that of pure rice wine. Among them, the AWY and De samples had more red and blue parts, indicating that their flavor substances were rich in composition and more prominent in characteristics. In contrast, the Ra and Ba samples had fewer red parts and more blue parts, indicating that although their flavor substances were more diverse than those of pure rice wine, some of the flavor substances were less abundant.

The results of the characterization of some volatile substances in different wine samples are shown in Table 3. Among the substances that can reflect the flavor components of wine, there are 7 alcohols, 4 aldehydes, 16 esters, 4 ketones, 2 alkenes, and 1 pyrazine. The presence of esters enriches the flavor of pineapple glutinous rice wine [29], and most of the ester compounds are derived from the esterification reaction during yeast fermentation [30]. Substances that can reflect the fruit flavor of pineapple glutinous rice wine, such as n-hexanol, n-nonanal, butyl acetate, ethyl propionate, and methyl acetate, were detected. Isoamyl acetate is a typical ester volatile component, with a strong banana-fruity aroma, which is the key characteristic marker to highlight the fruity and full-bodied flavor of pineapple glutinous rice wine. The differences in their contents could be distinguished by the color shades of the corresponding ingredient spots in Figure 5b and the relative contents in Table 3. The relative contents of substances in the De yeast and AWY samples were higher.

#### 3.8.2. Gallery Plot Fingerprint Analysis of Volatile Substances

To more objectively and accurately represent the differences in volatile substance content between different commercial yeast-fermented samples, the Gallery Plot was used to draw the volatile substance fingerprint profiles of pure rice wine and pineapple glutinous rice wine fermented with four commercial yeasts. The shades of red in the figure reflect the differences in volatile substance content. As shown in Figure 5c, the types of volatile substances varied greatly among different samples, and the red boxes indicate relatively high-content substances in the respective samples. The flavor substances characteristic of rice wine, including [31,32] isobutanol (with a special aroma), trans-2-hexenal (with fresh green leaf and fruity aromas), ethyl acetate (with a fruity-like aroma), isoamyl acetate (with a banana-like odor), methyl acetate (with a fruity aroma), and 2,6-dimethylpyrazine (with a burnt aroma), were common to different samples, and their relative contents were close to each other. In pure rice wine, n-nonanal (with rose and citrus aromas), n-propyl acetate (with an aromatic odor), and terpinolene (with a pinewood odor) were found to have higher contents. Ra wine samples contained high levels of sec-butanol (with a wine aroma), isobutyl acetate (with a fruity aroma), and β-pinene (with a turpentine odor). AWY wine samples had high levels of methyl isovalerate (with a fruity odor) and ethyl propionate (with a pineapple aroma). Ba samples had high levels of β-pinene (with a turpentine odor), methyl acetate (with a fruity odor), and butyl acetate (with a fruity aroma). De wine samples had higher levels of methyl isovalerate (with a fruity aroma), isovaleraldehyde (with an apple aroma), and ethyl propionate (with a pineapple aroma).

#### 3.8.3. Analysis of Characteristic Compounds

**Figure 6:**
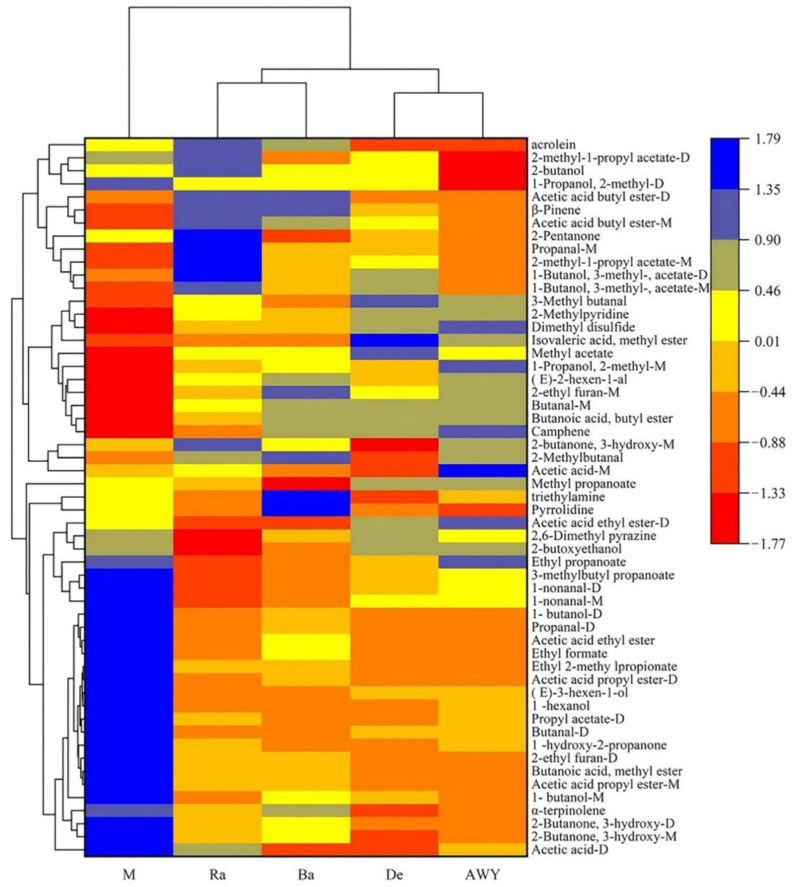
Heat map of changes in characteristic compounds of wine samples after fermentation with different commercial yeasts.

The results of the heat map depicting the variation in characteristic compounds in pure rice wine and pineapple glutinous rice wine are presented in Figure 6. Pure rice wine was independently clustered into one category. The remaining four types of pineapple rice wines were grouped into a second category, with the Ra and Ba samples forming one sub-category, and the De and AWY samples constituting another sub-category within this larger grouping.

The relative contents of alcohols in the five samples (M, Ra, Ba, De, and AWY) were 13.75%, 12.46%, 12.15%, 12.02%, and 10.34%, respectively. The relative content of aldehydes was 28.10%, 31.25%, 31.78%, 30.81%, and 32.44%; that of ketones was 3.66%, 2.04%, 2.22%, 1.66%, and 1.67%; the relative content of acids was 13.26%, 13.04%, 12.37%, 12.18%, and 13.17%; and the relative contents of esters were 21.06%, 21.65%, 20.33%, 22.55%, and 21.24%, respectively.

The flavor substances were clustered into three categories through analysis. The first category encompassed acrolein, isobutyl acetate, sec-butanol, isobutanol, butyl acetate, β-pinene, 2-pentanone, and isoamyl acetate. The second category included isovaleraldehyde, 2-methylpyridine, methyl isovalerate, methyl acetate, trans-2-hexenal, 2-ethylfuran, n-butyraldehyde, butyl butyrate, camphene, 3-hydroxy-2-butanone, and 2-methylbutanal. The third group consisted of diethylamine, tetrandrine, ethyl acetate, 2,6-dimethylpyrazine, ethyl propionate, isoamyl propionate, n-nonenal, n-butanol, ethyl formate, ethyl 2-methylpropionate, n-propyl acetate, trans-3-hexen-1-ol, n-hexanol, 2-butanone, and hydroxyacetone. These three groups of substances were separately clustered, indicating a strong correlation among the components within each group. Among them, n-hexanol, n-nonanal, butyl acetate, ethyl propionate, and methyl acetate were identified as the main flavor substances influencing the quality of pineapple glutinous rice wine.

#### 3.8.4. Analysis of Key Flavor Compounds in Pineapple Glutinous Rice Wine Based on OPLS-DA

Orthogonal Partial Least Squares Discriminant Analysis (OPLS-DA) is a supervised multivariate discriminant method based on orthogonal partial least squares, which decomposes the data matrix into predictive components and orthogonal components. It can more precisely represent the differences between individual samples and categorize them according to the relevance of different substances [33]. The variable importance projection (VIP) is commonly used to evaluate the importance of variables in the OPLS-DA model. Generally, the larger the VIP value, the more crucial the aroma substance is in differentiating pineapple glutinous rice wine samples. Variables with a VIP > 1.0 are considered to make a relatively high contribution to sample differentiation [34].

**Figure 7:**
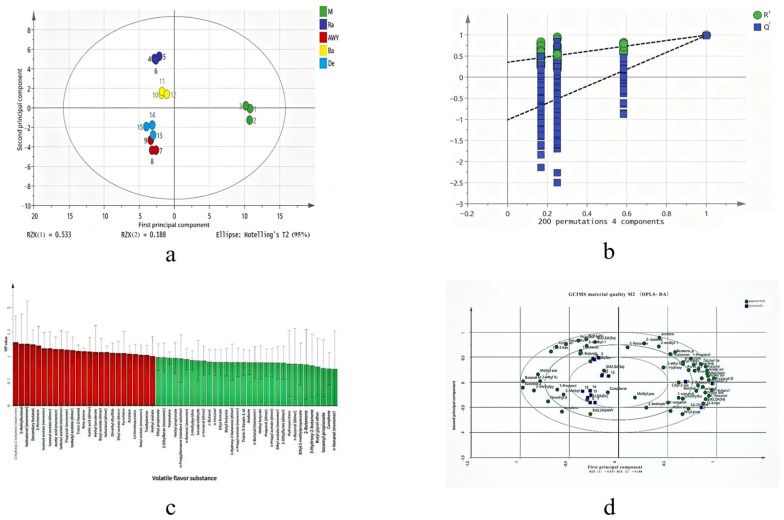
OPLS-DA plot of flavor compounds in wine samples fermented by different commercial yeasts: (**a**) OPLS-DA score plot; (**b**) OPLS-DA permutation test model (R^2^: model explanatory power, Q^2^: model predictive power); (**c**) VIP plot; (**d**) Biplot plot (X: independent variable, Y: categorical variable, t (corr.) (2): correlation coefficient between score value t and variables X and Y).

The volatile flavor substances in pineapple glutinous rice wine were selected for OPLS-DA analysis, as illustrated in Figure 7a–d.

The fitting index (R^2^x) of the independent variable in the OPLS-DA model for pure rice wine and pineapple glutinous rice wine samples was 0.930, the fitting index (R^2^y) of the dependent variable was 0.970, and the predictive index (Q^2^) of the model was 0.905. Since both R^2^ and Q^2^ were greater than 0.5, the results of the model fitting were deemed acceptable [35].

After 200 permutation tests (Figure 7b), the intercept between the Q^2^ regression line and the vertical axis was less than 0, indicating that the model was not overfitted and was thus valid for analyzing the flavor components of pure rice wine and pineapple glutinous rice wine. Based on VIP > 1 and *p* < 0.05, 25 key aroma substances were screened out (Figure 7c), including 3-hydroxy-2-butanone-monomer, 2-methylbutyraldehyde, isobutanol-monomer, sec-butanol, 2-pentanone, isoamyl acetate-monomer, isoamyl acetate-dimer, acetic acid-monomer, isobutyl acetate-monomer, propionaldehyde-monomer, isobutyl acetate-dimer, trans-2-hexenal, β-pinene, acetic acid-dimer, methyl isovalerate, butyl acetate-dimer, dimethyldisulfide, ethyl acetate-dimer, tetrahydropyrrole, acrolein, 2,6-dimethylpyrazine, butyl acetate-monomer, triethylamine, and methyl acetate. Isoamyl acetate, with typical banana and fruity aroma, is a core characteristic marker that highlights the fruity flavor of pineapple glutinous rice wine. These substances can be regarded as the key flavoring substances for differentiating between pure rice wine and pineapple glutinous rice wine.

As shown in Figure 7d, pure rice wine and the four yeast-fermented pineapple glutinous rice wines were distributed in three quadrants. The compounds that clustered together were the key flavor substances differentiating the samples, which were either significantly higher in the corresponding samples or only present in that specific sample. Pure rice wine was located in the fourth quadrant, with n-hexanol being the key differentiating flavor substance. Pineapple glutinous rice wines fermented by Ra and Ba yeasts were in the second quadrant, and n-butanol and 2-methylbutyraldehyde were their key differentiating flavor substances. Pineapple glutinous rice wines fermented by De and AWYs were in the third quadrant, with camphene and isovaleraldehyde serving as the key differentiating flavor substances. Based on these key flavor substances, effective differentiation among the five samples could be achieved.

## 4. Discussion

This study performed a comprehensive evaluation of the sensory quality, physicochemical indices, antioxidant properties, and volatile components of pineapple glutinous rice wine brewed with four commercial yeasts (Ra, Ba, De, and AWY). The results demonstrated that the 0.2 g/L De sample exhibited the most favorable quality development during fermentation. It achieved the highest relative contents of alcohol, flavonoids, and VC, accompanied by more harmonious color, balanced sensory profile, abundant volatile substances and appropriate total acid content, with total acid and total sugar maintained at an optimal level. Notably, the fermentation efficiency and sugar-to-alcohol conversion rate (70.62%) of 0.2 g/L De in this study were significantly higher than those of other fruit-flavored glutinous rice wines, along with richer volatile components, which fills the research gap in the screening of dedicated commercial yeasts for pineapple glutinous rice wine.

Based on GC-IMS and fingerprint analysis, n-hexanol, n-nonanal, butyl acetate, ethyl propionate, and methyl acetate were identified as the main flavor substances in pineapple glutinous rice wine. These five key aroma substances show a remarkable synergistic effect: n-hexanol and n-nonanal strengthen fresh fruity and floral aromas, butyl acetate and methyl acetate enrich the sweet fruit flavor, and ethyl propionate highlights the characteristic pineapple aroma, jointly forming a coordinated flavor with prominent pineapple aroma and soft rice wine taste. De shows the optimal performance mainly due to its high esterification enzyme activity, which can efficiently catalyze the production of ester compounds (relative ester content up to 22.55%), and its mild metabolic activity can better retain flavonoids and vitamin C, reducing the loss of antioxidant substances.

Comparative analysis of multiple physicochemical indices confirmed that the overall quality of pineapple glutinous rice wine fermented with 0.2 g/L De yeast was superior to that of Ra, Ba, and AWY. The wine obtained with De contained higher contents of esters, aldehydes, and other flavor substances, and displayed better fermentation performance, proving to be the most suitable yeast strain. The conclusion of “reducing fermentation costs” is supported by the fact that 0.2 g/L De has the highest sugar-to-alcohol conversion efficiency, shortens the effective fermentation time, and does not require additional additives to adjust the flavor, thereby reducing raw material and time costs in industrial production.

This study systematically characterizes the organic acid accumulation and flavor variation in pineapple glutinous rice wine during commercial yeast fermentation, providing valuable technical references for improving fermentation efficiency and reducing production costs in the industrial production of multi-flavored glutinous rice wines. Meanwhile, it establishes a solid theoretical basis for quality control and further development of pineapple glutinous rice wine. For practical industrial application, the optimized fermentation conditions obtained in this research can be directly applied to winery production to stabilize flavor consistency and lower trial-and-error costs in large-scale brewing. It should be acknowledged that the current study did not incorporate independent biological fermentation replicates, which is attributed to the highly standardized nature and low batch variability of commercial yeast products. Accordingly, the present results are exploratory, and future well-designed studies with sufficient biological replicates are recommended to consolidate these findings and promote their industrial implementation.

## Figures and Tables

**Table 1:** Color difference results of wine samples fermented with different commercial yeasts.

Species	L*	a*	b*	ΔE*
0.1 g/L Ra	8.33 ± 0.22 ^a^	27.80 ± 0.46 ^a^	11.50 ± 0.64 ^a^	31.22 ± 0.58 ^a^
0.2 g/L Ra	7.44 ± 0.25 ^b^	26.98 ± 0.41 ^a^	10.05 ± 0.15 ^b^	29.74 ± 0.36 ^b^
0.3 g/L Ra	8.62 ± 0.31 ^a^	27.28 ± 0.64 ^a^	11.58 ± 0.23 ^a^	30.86 ± 0.50 ^a^
0.1 g/L Ba	8.54 ± 0.27 ^a^	30.88 ± 0.52 ^a^	11.24 ± 0.26 ^a^	33.95 ± 0.52 ^a^
0.2 g/L Ba	7.84 ± 0.32 ^b^	15.37 ± 0.43 ^c^	10.80 ± 0.26 ^ab^	20.36 ± 0.28 ^c^
0.3 g/L Ba	7.72 ± 0.14 ^b^	23.55 ± 0.30 ^b^	10.50 ± 0.20 ^b^	26.92 ± 0.32 ^b^
0.1 g/L De	8.03 ± 0.14 ^c^	20.36 ± 0.88 ^a^	6.81 ± 0.08 ^b^	22.92 ± 0.75 ^a^
0.2 g/L De	13.41 ± 0.06 ^a^	4.14 ± 0.04 ^c^	14.30 ± 0.18 ^a^	20.04 ± 0.12 ^b^
0.3 g/L De	8.56 ± 0.13 ^b^	15.65 ± 0.19 ^b^	14.06 ± 0.41 ^a^	22.72 ± 0.36 ^a^
0.1 g/L AWY	10.92 ± 0.24 ^a^	28.38 ± 0.43 ^a^	9.36 ± 0.09 ^b^	31.82 ± 0.42 ^a^
0.2 g/L AWY	10.30 ± 0.16 ^b^	17.04 ± 0.59 ^c^	7.70 ± 0.20 ^c^	21.35 ± 0.56 ^c^
0.3 g/L AWY	6.26 ± 0.18 ^c^	25.08 ± 0.61 ^b^	12.84 ± 0.15 ^a^	28.86 ± 0.52 ^b^

Note: Different labeled letters in the same column of data indicate significant differences (*p* < 0.05).

**Table 2:** Correlation analysis results.

Indices	Yeast Addition	Total Sugar Content	Vitamin C	Total Flavonoids
Yeast addition	1			
Total sugar content	−0.546 **	1		
Vitamin C	0.403 *	−0.660 **	1	
Total flavonoids	0.476 **	−0.724 **	0.912 **	1

Note: * indicates significant correlation (*p* < 0.05); ** indicates a highly significant correlation (*p* < 0.01).

**Table 3:** Qualitative results of some volatile substances in wine samples fermented by different commercial yeasts.

	Compound Name	Relative Content (%)					Odor Description	Comment
		M	Ra	Ba	De	AWY		
Alcohols	Butanol	0.54	0.3	0.41	0.35	0.32	Special aroma	Monomer
	Butanol	0.20	0.05	0.08	0.08	0.05	Special aroma	Dimer
	Isobutyl alcohol	11.17	10.39	9.98	9.98	8.30	Special aroma	Dimer
	Isobutyl alcohol	0.97	1.17	1.21	1.14	1.35	Special aroma	Monomer
	sec-Butyl alcohol	0.35	0.42	0.33	0.35	0.17	Wine aroma	
	Hexyl alcohol	0.31	0.09	0.09	0.09	0.10	Fruity aroma	
	trans-3-Hexen-1-ol	0.21	0.04	0.05	0.05	0.05	Aroma of green leaves	
Aldehydes	n-Nonanal	0.51	0.13	0.19	0.25	0.27	Rose and citrus aromas	Dimer
	n-Nonanal	2.33	1.13	1.37	1.83	1.85	Rose and citrus aromas	Monomer
	Isovaleric aldehyde	0.22	0.43	0.28	0.52	0.50	Aroma of apple	
	trans-2- Hexenal	23.10	26.95	27.45	25.98	27.53	Aroma of green leaves, Fruity aroma	
Esters	Isoamyl acetate	1.54	2.12	1.80	2.01	1.69	Banana flavor	Monomer
	Isoamyl acetate	1.42	3.03	1.74	2.57	1.47	Banana flavor	Dimer
	Butyl acetate	0.03	0.16	0.15	0.11	0.07	Fruity aroma	Monomer
	Butyl acetate	0.02	0.04	0.04	0.02	0.02	Fruity aroma	Dimer
	Methyl isovalerate	0.02	0.05	0.04	0.15	0.12	Fruity aroma	
	Isobutyl acetate	0.25	0.40	0.28	0.32	0.27	Fruity aroma	Monomer
	Isobutyl acetate	0.27	0.33	0.14	0.20	0.04	Fruity aroma	Dimer
	n-Propyl acetate	1.06	0.07	0.11	0.05	0.05	Aromatic scent	
Esters	Ethyl propionate	0.30	0.06	0.13	0.14	0.28	Pineapple aroma	
	Ethyl acetate	6.83	6.02	6.12	7.02	7.18	Fruity aroma	Dimer
	Ethyl acetate	0.53	0.33	0.38	0.32	0.34	Fruity aroma	Monomer
	Ethyl formate	0.70	0.25	0.35	0.21	0.23	Aromatic scent	
	Isoamyl propionate	0.19	0.08	0.09	0.11	0.13	Aroma of apple	
	Methyl butyrate	0.23	0.05	0.04	0.03	0.03	Aroma of apple	
	Ethyl 2-methylpropionate	0.34	0.04	0.04	0.03	0.03	Fruity aroma	
	Methyl acetate	1.09	1.56	1.52	1.68	1.53	Fruity aroma	
Ketones	3-Hydroxy-2-butanone	1.08	0.77	0.86	0.61	0.68	Aroma of cream	Monomer
	3-Hydroxy-2-butanone	0.68	0.33	0.40	0.23	0.25	Aroma of cream	Dimer
	Hydroxy acetone	0.13	0.07	0.06	0.06	0.07	Special aroma	
	2-Butanone	0.07	0.02	0.01	0.02	0.02	Special aroma	
Others	β-Pinene	0.03	0.18	0.19	0.11	0.05	Turpentine scent	
	Terpinolene	1.39	0.87	1.17	0.45	0.65	Pine wood scent	
	2,6-Dimethylpyrazine	2.81	2.21	2.49	2.77	2.70	Scorched aroma	

Note: M refers to pure glutinous rice wine, control group.

## Data Availability

The data presented in this study are available on request from the corresponding author.

## Abbreviations

The following abbreviations are used in this manuscript:

Ra	La Raffinée
Ba	La Bayanus
De	La Délicieuse
AWY	Angel wine yeast
VC	Vitamin C
GC-IMS	gas chromatography–ion mobility spectrometry
RIs	retention indices
CO_2_	Carbon dioxide
L*	the lightness of color
a*	related to the red component
b*	related to the yellow component
ΔE*	the color difference
OPLS-DA	Orthogonal Partial Least Squares Discriminant Analysis
VIP	variable importance projection
